# Natural Killer cells present in gut mucosa as potential ADCC effector cells

**DOI:** 10.1186/1742-4690-9-S2-P188

**Published:** 2012-09-13

**Authors:** M Sips, G Sciaranghella, N Tong, D Kwon, P Brouckaert, G Alter

**Affiliations:** 1 Ghent University, Ghent, Belgium; 2 Ragon Institute, USA, Charlestown, MA, USA

## Background

Data from the RV144 HIV vaccine trial showed a moderate protection from HIV infection in the absence of neutralizing antibodies or CTL activity. In contrast, all vaccinees mounted robust HIV-specific binding antibodies, that may have provided some level of protection through their capacity to recruit antibody dependent cellular cytotoxicity (ADCC). The capacity of an antibody to recruit ADCC relies on its ability to interact with the Fc-receptor, FCγRIII (CD16), expressed on Natural Killer (NK) cells. However, little is known about innate immune effector cells present within mucosa, and whether they have the capacity to be harnessed by ADCC inducing antibodies should they be elicited by a vaccine. Here we hypothesized that abundant numbers of CD16+ NK cells line the gut mucosa, providing a robust effector arm that could be harnessed by ADCC inducing antibodies.

## Methods

The frequency and function of CD16+ cells in the colon was assessed by flow cytometry following enzymatic digestion of intestinal resections from HIV-uninfected subjects.

## Results

Significantly fewer NK cells were found in colon resections compared to peripheral blood of healthy controls (median: 10.40% vs. 18.80%, p=0.0062, respectively). Furthermore, while both CD56^bright^ immunoregulatory and CD56^dim^ cytolytic NK cells able to mediate ADCC were present in the gut, the frequency of these 2 subsets was altered compared to the blood. Moreover, fewer NK cells expressed NKp46, NKG2A, KIR, CD8, perforin, and importantly CD16 in the gut compared to the blood. However, gut NK cells demonstrated similar to blood cytolytic activity upon stimulation.

## Conclusion

Taken together, these data suggest that ADCC inducing antibodies present within the gut mucosa may likely recruit the antiviral activities of NK cells. Greater emphasis should be placed on developing innate immune recruiting antibody assays that measure the capacity of antibodies to recruit the antiviral activity of innate immune cells present within mucosal membranes where transmission occurs.

